# Integrative transcriptomic analysis reveals the molecular responses of tobacco to magnesium deficiency

**DOI:** 10.3389/fpls.2024.1483217

**Published:** 2024-11-25

**Authors:** Tingmin Liang, Jinbin Lin, Shengxin Wu, Rongrong Ye, Mengyu Qu, Rongrong Xie, Yingfeng Lin, Jingjuan Gao, Yuemin Wang, Yuqin Ke, Chunying Li, Jinping Guo, Jianjun Lu, Weiqi Tang, Songbiao Chen, Wenqing Li

**Affiliations:** ^1^ Institute of Tobacco Sciences, Fujian Provincial Tobacco Monopoly Bureau, Fuzhou, China; ^2^ Ministerial and Provincial Joint Innovation Centre for Safety Production of Cross-Strait Crops, College of Geography and Oceanography, Minjiang University, Fuzhou, China; ^3^ College of Agriculture, Fujian Agriculture and Forestry University, Fuzhou, China; ^4^ College of Plant Protection, Fujian Agriculture and Forestry University, Fuzhou, China; ^5^ International Magnesium Institute, Fujian Agriculture and Forestry University, Fuzhou, China; ^6^ College of Life Science, Fujian Agriculture and Forestry University, Fuzhou, China

**Keywords:** tobacco, magnesium, transcriptome analysis, Mg^2+^ transporter, Mg^2+/H+^ exchanger, photosynthesis, antioxidant response

## Abstract

**Introduction:**

Magnesium (Mg) is a crucial macronutrient for plants. Understanding the molecular responses of plants to different levels of Mg supply is important for improving cultivation practices and breeding new varieties with efficient Mg utilization.

**Methods:**

In this study, we conducted a comprehensive transcriptome analysis on tobacco (*Nicotiana tabacum* L.) seedling leaves to investigate changes in gene expression in response to different levels of Mg supply, including Mg-deficient, 1/4-normal Mg, normal Mg, and 4×-normal Mg, with a particular focus on Mg deficiency at 5, 15 and 25 days after treatment (DAT), respectively.

**Results:**

A total of 11,267 differentially expressed genes (DEGs) were identified in the Mg-deficient, 1/4-normal Mg, and/or 4×-normal Mg seedlings compared to the normal Mg seedlings. The global gene expression profiles revealed potential mechanisms involved in the response to Mg deficiency in tobacco leaves, including down-regulation of genes–two DEGs encoding mitochondria-localized NtMGT7 and NtMGT9 homologs, and one DEG encoding a tonoplast-localized NtMHX1 homolog–associated with Mg trafficking from the cytosol to mitochondria and vacuoles, decreased expression of genes linked to photosynthesis and carbon fixation at later stages, and up-regulation of genes related to antioxidant defenses, such as *NtPODs*, *NtPrxs*, and *NtGSTs*.

**Discussion:**

Our findings provide new insights into the molecular mechanisms underlying how tobacco responds to Mg deficiency.

## 1 Introduction

Magnesium (Mg) is an essential macronutrient for plants, vital for photosynthesis, enzyme activation, protein synthesis, and nucleotide metabolism (Cowan, 2002; Chaudhry et al., 2021). As the central atom in the chlorophyll molecule, Mg plays a crucial role in various processes such as chlorophyll biosynthesis, photosynthetic metabolism, and CO_2_ assimilation (Verbruggen and Hermans, 2013). Additionally, Mg acts as a cofactor and allosteric modulator for a variety of enzymes, including carboxylases, phosphatases, protein kinases, RNA polymerases, and ATPases (Shaul, 2002), therefore influencing various physiological, biochemical, and cellular processes for plant growth and development (Cakmak and Kirkby, 2008).

Mg is one of the most abundant elements in the Earth’s crust. However, the majority of soil Mg (90-98%) is incorporated into various minerals and is not directly available to plants (Senbayram et al., 2015). The form of Mg that plant can absorb is Mg^2+^. Due to its small ionic radius but large hydrated radius, Mg^2+^ binds weakly to negatively charged soil colloids and is easily leached from acidic and sandy soils (Verbruggen and Hermans, 2013), resulting in Mg deficiency in agricultural lands worldwide (Maathuis, 2009). Mg deficiency reduces chlorophyll biosynthesis, causes photooxidative damage, and impairs the phloem loading of photoassimilates in plants (Chaudhry et al., 2021). Common morphological symptoms of Mg deficiency in plants include growth retardation and interveinal leaf chlorosis (Marschner and Cakmak, 1989; Cakmak and Kirkby, 2008). Ultimately, Mg deficiency leads to significant reductions in crop yield and quality (Moss and Higgins, 1974; Cakmak, 2013).

While Mg deficiency has been a prevalent issue in agriculture, it has gain significant attention in recent decades (Hauer-Jákli and Tränkner, 2019). In recent years, an increasing number of studies have focused on investigating the physiological and molecular mechanisms underlying plant responses to Mg deficiency (Ishfaq et al., 2022; Tang et al., 2022; Deng et al., 2023; Wang et al., 2023). However, the detailed mechanism remains incompletely understood. Tobacco (*Nicotiana tabacum* L.) is an important model in plant biology and is a significant economic crop. Mg is vital for the growth and development of tobacco. Mg deficiency is prevalent in soils suited for tobacco cultivation, leading to decreased yield and quality (Liu et al., 1998; Li et al., 2022). Several studies have demonstrated that the proper application of Mg fertilizers enhances the growth, development, yield, and leaf quality of flue-cured tobacco (Liu et al., 1998; Xu et al., 2011; Li et al., 2022, 2023). However, there is limited knowledge regarding the molecular response mechanism of tobacco to Mg deficiency. In the present study, we performed an RNA-Seq analysis on tobacco seedlings grown under Mg deficiency and at three different levels of Mg supply. Our results revealed dynamic changes in gene expression in response to Mg deficiency in tobacco leaves, including down-regulation of genes involved in Mg trafficking from the cytosol to mitochondria and vacuoles, decreased expression of genes related to photosynthesis and carbon fixation at later stages, and up-regulation of genes associated with antioxidant defenses.

## 2 Materials and methods

### 2.1 Plant materials and growth conditions

Tobacco (*Nicotiana tabacum* L. cv. CB-1) seedlings were first germinated in soil trays and the grown under standard conditions for approximately 5-6 weeks, until they reached the seven-leaf stage. Subsequently, tobacco seedlings were transferred from soil trays to 1000 mL plastic boxes filled with a modified Hoagland nutrient solution. The nutrient solution consisted of the following components: 4.66 mM Ca(NO_3_)_2_·4H_2_O, 1.41 mM KH_2_PO_4_, 4.98 mM KNO_3_, 1.99 mM MgSO_4_·7H_2_O, 0.10 mM FeSO_4_·7H_2_O, 0.10 mM EDTA-2Na, 46.26 μM H_3_BO_3_, 9.10 μM MnCl_2_·4H_2_O, 0.77 μM ZnCl_2_, 0.41 μM CuCl_2_·2H_2_O, and 0.13 μM Na_2_MoO_4_·2H_2_O (Lu et al., 2023).

After a pre-culture of one week in growth chambers under a 12-hour light at 25°C/12-hour dark at 20°C, the well-grown tobacco seedlings were transferred to new plastic boxes and cultured in the modified Hoagland nutrient solutions with the Mg concentrations of Mg-deficient (0 mM), 1/4-normal Mg (0.50 mM), normal Mg (1.99 mM), and 4×-normal Mg (7.96 mM) (hereafter designated as Mg0, Mg1/4, Mg1, and Mg4), respectively. Tobacco seedlings were maintained in the growth chambers under the same light-dark cycle and temperature conditions as during the pre-culture phase. The liquid solutions in the plastic boxes were completely replaced every three days to ensure fresh nutrients for the seedlings. Each treatment group consisted of three replicates, with each replicate containing three seedlings.

### 2.2 Measurement of physiological traits

The first fully expanded leaf of tobacco seedlings grown under different levels of Mg supply was sampled at 25 days after treatment (DAT). A total of 14 physiological parameters were measured, including the contents of Mg, chlorophyll a (Chla), chlorophyll b (Chlb), carotenoids (Car), soluble proteins (SP), and hydrogen peroxide (H_2_O_2_); the activities of ribulose-1,5-bisphosphate carboxylase (RuBPCase), acid invertase (AI), neutral invertase (NI), nitrate reductase (NR), superoxide dismutase (SOD), catalase (CAT), and peroxidase (POD); root vitality; as well as cell membrane permeability (CMP). Each experiment had three biological replicates.

The measurements were conducted following established methods as previously described (Lu et al., 2023). Specifically, the content of Mg was determined using inductively coupled plasma optical emission spectroscopy (ICP-OES) system (iCAP 7000 Series, Thermo Fisher Scientific, USA) (Musharraf et al., 2012). The contents of Chla, Chlb, and Car were measured using a spectrophotometer, as per the protocol by Lichtenthaler and Buschmann (2001); SP and H_2_O_2_ levels were quantified following the procedures described by Anderson et al. (1995) and by Clemensson-Lindell (1994), respectively; RuBPCase activity was measured following the procedure described by Leech et al. (1985), while AI, NI, NR, SOD, CAT, and POD activities were assessed using Zou’s methodologies (Zou, 2000); root vitality was determined using the triphenyltetrazolium chloride (TTC) method (Clemensson-Lindell, 1994); lastly, CMP of leaves was determined through electrolyte leakage, as described by Palta and Stadelmann (1997).

### 2.3 RNA-seq analysis

The first fully expanded leaf of tobacco seedlings grown in nutrient solutions with different levels of Mg supply was collected at 5, 15, and 25 DAT, respectively, for RNA-Seq analysis. Each treatment consisted of three independent biological replicates, with one seedling per replicate. Total RNA was extracted from finely ground leaf samples using TRIzol reagent (Thermo Fisher Scientific, China). The extracted RNA was treated with RNase-free DNase I (Thermo Fisher Scientific, China) to eliminate genomic DNA contamination. The RNA samples were then subjected to RNA-Seq analysis at Novogene, Beijing, China, using an Illumina Novaseq platform.

The resulting sequencing data was processed and were mapped to the reference genome of tobacco available at https://solgenomics.net/organism/Nicotiana_tabacum/genome, using the HISAT2 v2.0.5 program (Kim et al., 2015). The expression level of each gene was quantified based on the fragments per kilobase of transcript per million mapped reads (FPKM) value (Trapnell et al., 2010). Differential expression analysis was performed using the DESeq2 package in R platform (Love et al., 2014). Genes with a |log2 FC| ≥ 1 and the false discovery rate (FDR) ≤ 0.05 were considered as differentially expressed genes (DEGs).

### 2.4 Gene ontology and Kyoto encyclopedia of genes and genomes analysis

The identified DEGs were subjected to GO analysis using the GOseq R packages (Young et al., 2010), and to KEGG pathway enrichment analysis using the KEGG Orthology-based Annotation System (KOBAS) software (Xie et al., 2011). GO terms or KEGG pathways with p ≤ 0.05 were considered for further assessment in this study.

### 2.5 Real-time quantitative RT-PCR

Total RNAs were extracted from tobacco leaves using the TransZol Up kit (TransGen Biotech, China) and were treated with RNase-free DNase I (Takara, China) to eliminate any contaminating DNA. First-strand cDNA synthesis was then performed using a HiScript II 1st Strand cDNA Synthesis Kit (Vazyme, China). Quantitative real-time PCR (qRT-PCR) reactions were carried out on a CFX Connect Real-Time System (BIO-RAD, USA) with a SYBR qPCR Master Mix (Vazyme, China). Three replications were conducted for each sample. Internal control tests were conducted with the tobacco *EF-1α* gene (Schmidt and Delaney, 2010). Relative expression values were calculated using the 2^-ΔΔCT^ method (Livak and Schmittgen, 2001). The transcriptional profiles of 11 DEGs involved in Mg distribution and antioxidative regulation were validated using qRT-PCR. The specific primers used for the qRT-PCR analysis can be referenced in **Supplementary Table S1**.

### 2.6 Subcellular localization

The open reading fragment (ORF) sequences of the target genes were obtained from the Solanaceae Genomics Network (https://solgenomics.net/ftp/genomes/Nicotiana_tabacum/edwards_et_al_2017/). Subcellular localization constructs for the homologous NtMGT7 (Nitab4.5_0003331g0150) and NtMGT9 (Nitab4.5_0000436g0030), and the homologous NtMHX1 (Nitab4.5_0005805g0040), were created by amplifying and inserting the ORFs of these genes into pCS-NGFP (Qu et al., 2021) to fuse with the *GFP* gene. The resulting constructs were then introduced into the *Agrobacterium tumefaciens* strain GV3101. The GV3101 bacteria carrying these GFP-fusion constructs, a mitochondria ScCOX4-DsRed marker construct or a tonoplast Osγ-TIP-DsRed marker construct (Chen et al., 2019) were cultured in liquid yeast extract peptone media supplemented with kanamycin (50 μg/ml) and rifampicin (50 μg/ml). Suspensions of the GV3101 bacteria containing the GFP-fusion constructs were co-infiltrated with the DsRed marker constructs into leaves of 4-week-old *Nicotinana benthamiana* plants grown in a growth chamber at 25°C under a 16/8 h light/dark cycle. The infiltrated plants were maintained in darkness at 25°C for 3 days. The infiltrated leaf tissues were collected and used to isolate protoplasts. Fluorescence microscopy was conducted on the *N. benthamiana* protoplasts using a Leica DMi8 Laser Scanning Confocal microscope (STELLARIS 5, Leica, Germany) with Excitation/emission wavelengths 488/535 nm for green fluorescence, and 552/610 nm for red fluorescence.

## 3 Results

### 3.1 Mg deficiency inhibits growth of tobacco seedlings

**Figure 1:**
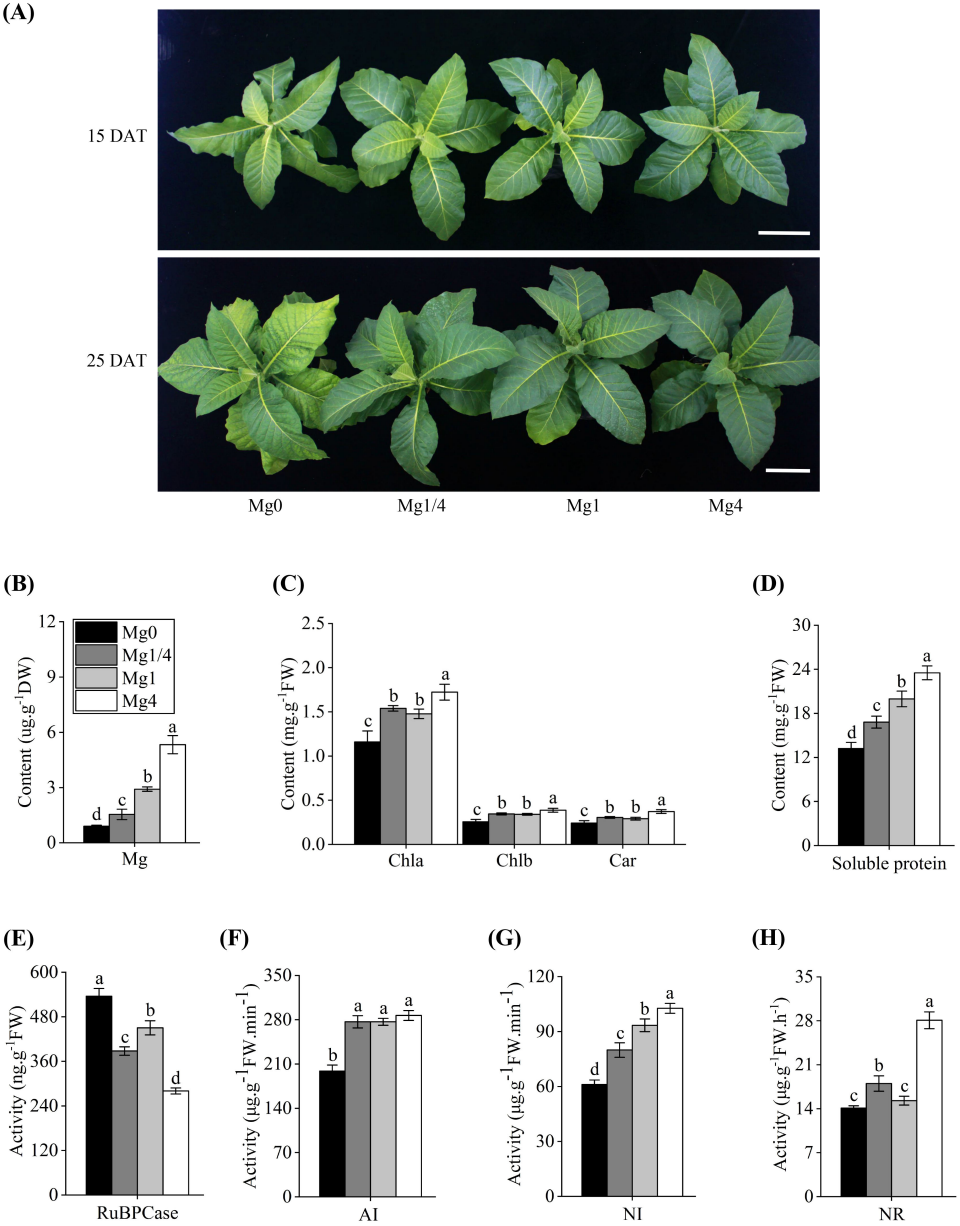
Effects of different Mg supplies on growth and physiological parameters of tobacco seedlings. **(A)** Phenotype of tobacco seedlings grown under different Mg supply levels at 15 and 25 DAT (days after treatment), respectively. Scale bars = 10 cm. Mg0, 0 mM Mg; Mg1/4, 0.50 mM Mg; Mg1, 1.99 mM Mg; Mg4, 7.96 mM Mg. **(B)** Magnesium (Mg) content in leaves of tobacco seedlings grown under different Mg supplies at 25 DAT. **(C)** Contents of chlorophyll a (Chla), chlorophyll b (Chlb), and carotenoids (Car) in leaves of tobacco seedlings grown under different Mg supplies at 25 DAT. **(D)** Concentration of soluble proteins (SP) in leaves of tobacco seedlings grown under different Mg supplies at 25 DAT. **(E)** Content of RuBPCase in leaves of tobacco seedlings grown under different Mg supplies at 25 DAT. **(F-H)** Activities of acid invertase (AI), neutral invertase (NI), and nitrate reductase (NR) in leaves of tobacco seedlings grown under different Mg supplies at 25 DAT. Different letters (a, b, c, d) above the columns indicate statistical differences (p < 0.05).

Tobacco seedlings were grown in modified Hoagland’s nutrient solutions supplied with four different levels of Mg (Mg0, Mg1/4, Mg1, and Mg4, respectively). At 5 DAT, no significant morphological differences were observed among the seedlings (data not shown). However, by 15 DAT, the Mg0 seedlings showed slight growth stunting, and by 25 DAT, they exhibited symptoms of leaf chlorosis and curling (**Figure 1A**). In contrast, the Mg1/4, Mg1, and Mg4 seedlings did not show noticeable morphological differences at either 15 or 25 DAT (**Figure 1A**).

At 25 DAT, the Mg content and eight physiological traits related to photosynthesis, carbon, and nitrogen metabolism in leaves of tobacco seedlings grown under different Mg levels were assessed. The Mg content in the Mg0 seedlings were notably lower compared to that in the Mg1/4, Mg1, and Mg4 seedlings (**Figure 1B**). The contents of three essential photosynthetic pigments (Chla, Chlb, and Car) in the Mg0 seedlings were significantly lower compared to those in the Mg1/4, Mg1, and Mg4 seedlings (**Figure 1C**). Similarly, the content of SP, an important osmoregulatory substance, was notably reduced in the Mg0 seedlings than in the Mg1/4, Mg1, and Mg4 seedlings (**Figure 1D**). In contrast, the activity of RuBPCase, a key C3 enzyme responsible for carbon fixation, was markedly higher in the Mg0 seedlings compared to the Mg1/4, Mg1, and Mg4 seedlings (**Figure 1E**). Furthermore, the activities of AI and NI, both involved in carbon metabolism, were significantly reduced in the Mg0 seedlings compared to the Mg1/4, Mg1, and Mg4 seedlings (**Figures 1F, G**). Similarly, the activity of NR, a crucial enzyme in nitrogen metabolism, was lower in the Mg0 seedlings than in the Mg1/4 and Mg4 seedlings (**Figure 1H**). These results demonstrated that a deficiency of Mg causes severe physiological disorders and inhibits the growth of tobacco seedlings.

### 3.2 Transcriptome profiling of genes showing differential expression in response to different levels of Mg supply

**Figure 2:**
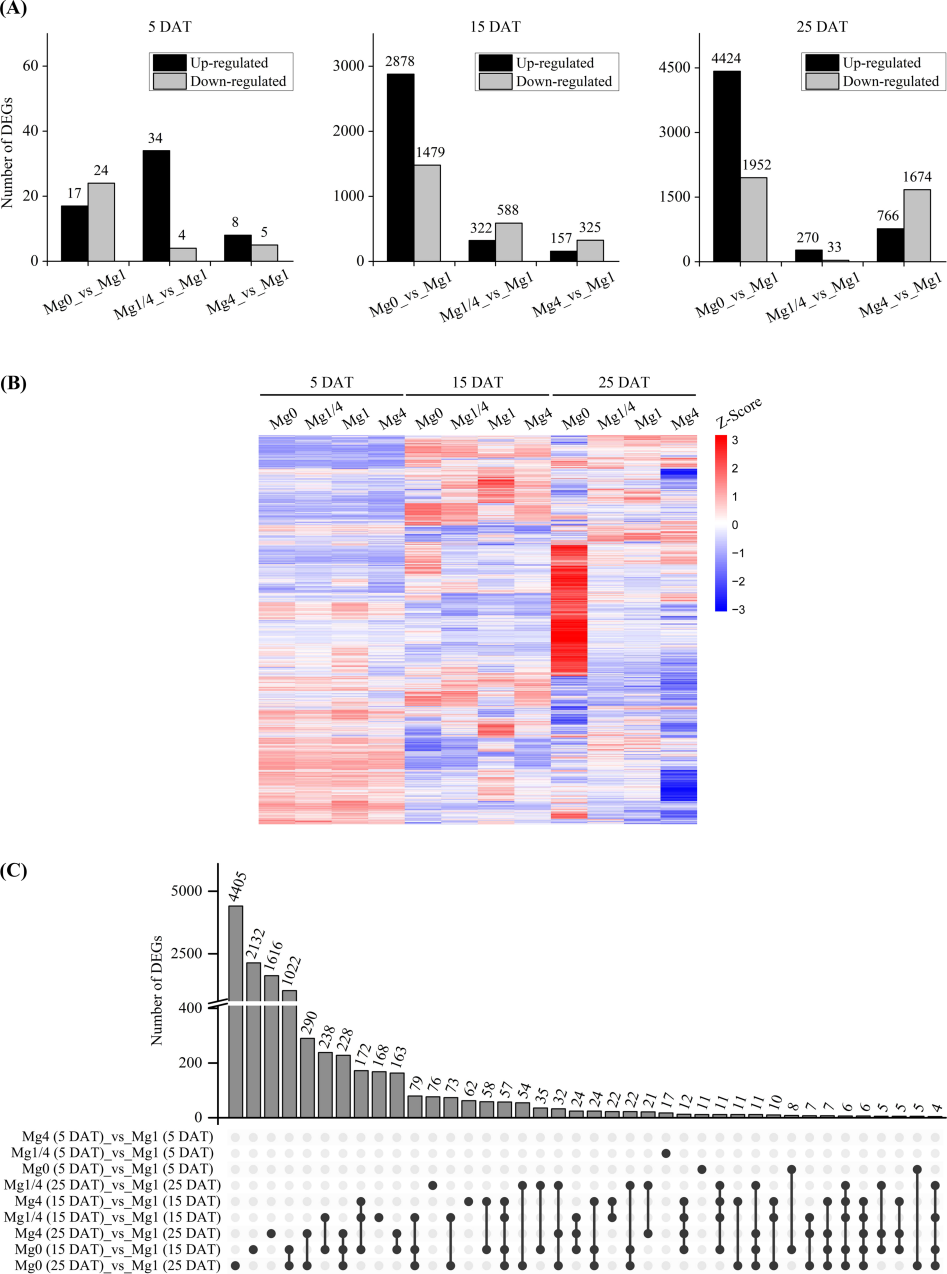
Overview of the differentially expressed genes (DEGs) in leaves of tobacco seedlings in response to different Mg supplies. **(A)** Number of the up-regulated and down-regulated DEGs in Mg0, Mg1/4, and Mg4 in comparison to Mg1 at 5, 15, and 25 DAT, respectively. **(B)** Heatmap showing the differential expression levels of the identified DEGs in leaves of tobacco seedlings grown under different Mg supplies at 5, 15, and 25 DAT, respectively. **(C)** Upset diagram showing the numbers of the DEGs specific or common in leaves of tobacco seedlings grown under different Mg supplies at 5, 15, and 25 DAT, respectively. Mg0, 0 mM Mg; Mg1/4, 0.50 mM Mg; Mg1, 1.99 mM Mg; Mg4, 7.96 mM Mg.

To investigate the molecular responses of tobacco plants to different levels of Mg supply, leaves from the Mg0, Mg1/4, Mg1, and Mg4 seedlings were collected at 5, 15, and 25 DAT, respectively, for RNA-Seq analysis. A total of 11,267 DEGs were identified in the Mg0, Mg1/4, and/or Mg4 seedlings compared to the Mg1 seedlings (**Supplementary Table S2**). While only a small number of DEGs were identified at 5 DAT (41 in Mg0, 38 in Mg1/4, and 13 in Mg4), a significantly higher number of DEGs were identified at 15 DAT (4,357 in Mg0, 910 in Mg1/4, and 482 in Mg4), and 25 DAT (6,376 in Mg0, 303 in Mg1/4, and 2,440 in Mg4), respectively (**Figure 2A** **;** **Supplementary Table S3**). Heatmap analysis revealed similar transcriptomic profiles for the Mg0, Mg1/4, Mg1, and Mg4 seedlings at 5 DAT, but more distinct differences emerged at 15 and 25 DAT, especially between the Mg0 seedlings and the other groups (**Figure 2B**).

Additionally, an upset analysis was conducted on the DEGs identified in the Mg0, Mg1/4, and/or Mg4 seedlings compared to the Mg1 seedlings to gain deeper insights into the regulation patterns of these DEGs in tobacco seedlings grown under various Mg levels over different growth durations (**Figure 2C**). For example, approximately 2,132 and 4,405 DEGs were specifically regulated in the Mg0 seedlings at 15 and 25 DAT, respectively, while 1,022 DEGs were co-regulated in the Mg0 seedlings at both 15 DAT and 25 DAT. In contrast, only 11 DEGs were specifically regulated in the Mg0 seedlings at 5 DAT, with eight DEGs co-regulated at both 5 DAT and 15 DAT, and five DEGs co-regulated at both 5 DAT and 25 DAT (**Figure 2C**). The results highlight that a significant number of genes were markedly induced in tobacco seedlings in response to Mg deficiency after 15 days of growth, with certain DEGs showing relatively long-term regulation.

### 3.3 DEGs involved in Mg distribution

**Figure 3:**
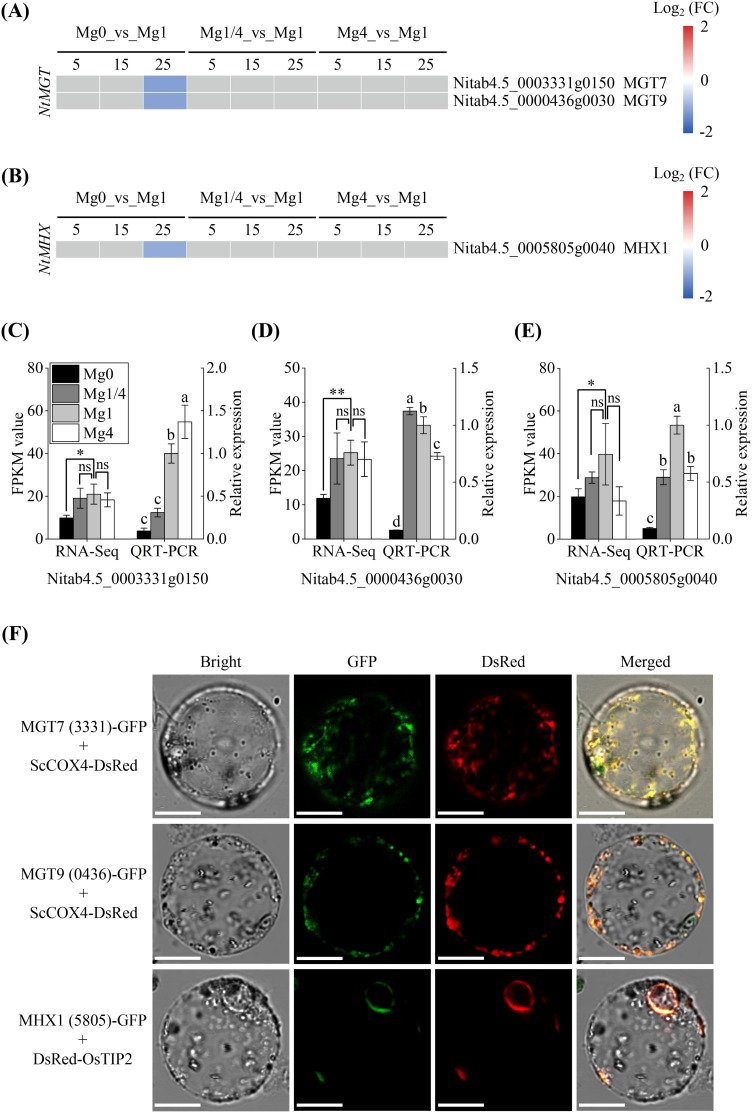
Expressional changes of DEGs involved in Mg distribution in tobacco in response to different Mg supplies. **(A, B)** Heatmap showing the differential expression patterns of two Mg^2+^ transporters (MGTs) DEGs and one Mg^2+^/H^+^ exchanger (MHX) DEG, respectively, at 5, 15, and 25 DAT. Grey blocks indicate that the genes were not detected as DEGs by RNA-Seq. Mg0, 0 mM Mg; Mg1/4, 0.50 mM Mg; Mg1, 1.99 mM Mg; Mg4, 7.96 mM Mg. DAT, days after treatment. **(C-E)** qRT-PCR validation of three DEGs *NtMGT7* (*Nitab4.5_0003331g0150*), *NtMGT9* (*Nitab4.5_0000436g0030*), and *NtMHX1* (*Nitab4.5_0005805g0040*) in leaves of the Mg0, Mg1/4, Mg1, and Mg4 seedlings at 25 DAT. The tobacco *EF-1α* gene was used as an internal control. Significance levels *p < 0.05, **p < 0.01, ns, not significant. Different letters (a, b, c, d) above the columns indicate statistical differences (p < 0.05). **(F)** Subcellular localization of NtMGT7 (Nitab4.5_0003331g0150), NtMGT9 (Nitab4.5_0000436g0030), and NtMHX1 (Nitab4.5_0005805g0040) in *N. benthamiana* protoplasts. Scale bars = 10 μm. MGT7 (3331)-GFP, NtMGT7 (Nitab4.5_0003331g0150)-GFP fusion; MGT9 (0436)-GFP, NtMGT9 (Nitab4.5_0000436g0030)-GFP fusion; MHX1 (5805)-GFP, NtMHX1 (Nitab4.5_0005805g0040)-GFP fusion; ScCOX4-Red, mitochondria ScCOX4-DsRed marker; DsRed-OsTIP2, tonoplast Osγ-TIP-DsRed marker.

Plants have developed mechanisms to transport and distribute Mg to maintain optimal cellular levels (Tang and Luan, 2017). Mg^2+^ transporters (MGTs) play essential roles in Mg uptake, transport and distribution (Yan et al., 2018). Among the DEGs identified, one *MGT7* homologous gene (*Nitab4.5_0003331g0150*) and one *MGT9* homologous gene (*Nitab4.5_0000436g0030*) were found to be down-regulated in the Mg0 seedlings at 25 DAT (**Figure 3A**). Mg^2+^/H^+^ exchanger (MHX) has been shown to facilitate Mg^2+^ influx into the vacuole (Shaul et al., 1999). A homologous *MHX1* gene (*Nitab4.5_0005805g0040*) was also identified as a DEG that was down-regulated in the Mg0 seedlings at 25 DAT (**Figure 3B**). To validate the transcriptome results, qRT-PCR analysis was conducted on the homologous *NtMGT7*, *NtMGT9*, and *NtMHX1* gene in the Mg0, Mg1/4, Mg1, and Mg4 seedlings at 25 DAT. The results indicated a significant down-regulation of all three genes in the Mg0 seedlings, consistent with the RNA-Seq data (**Figures 3C-E**).

The subcellular localization of MGTs and MHX is closely associated with their roles in the biological processes occurring within respective organelles. To investigate the subcellular localization of the homologous NtMGT7, NtMGT9 and NtMHX1 proteins, transient expression of the *NtMGT7*-*GFP*, *NtMGT9-GFP*, and *NtMHX1-GFP* fusions was conducted in *N. benthamiana* cells. The results demonstrated that the green fluorescence of NtMGT7-GFP, and NtMGT9-GFP co-localized with the mitochondria ScCOX4-DsRed marker (**Figure 3F**), indicating that both NtMGT7 and NtMGT9 are localized in the mitochondria. On the other hand, the green fluorescence of NtMHX1*-*GFP merged with the tonoplast Osγ-TIP-DsRed marker (**Figure 3F**), suggesting that NtMHX1 is localized to the vacuole membrane. These observations provide insights into the specific subcellular locations of NtMGT7, NtMGT9 and NtMHX1 and their respective roles within the cell.

### 3.4 DEGs involved in photosynthesis

To further understand the molecular mechanisms underlying tobacco’s response to different levels of Mg supply, a KEGG analysis was conducted on the DEGs from the Mg0, Mg1/4, and Mg4 seedlings compared to the Mg1 seedlings. The analysis revealed that both the up- and down-regulated DEGs in the Mg0, Mg1/4, and Mg4 seedlings were enriched in diverse pathways, particularly at 15 and 25 DAT. Significantly, multiple KEGG pathways associated with photosynthesis were prominently highlighted, including photosynthesis, photosynthesis-antenna proteins, and carbon fixation in photosynthetic organisms (**Supplementary Table S4**).

**Figure 4:**
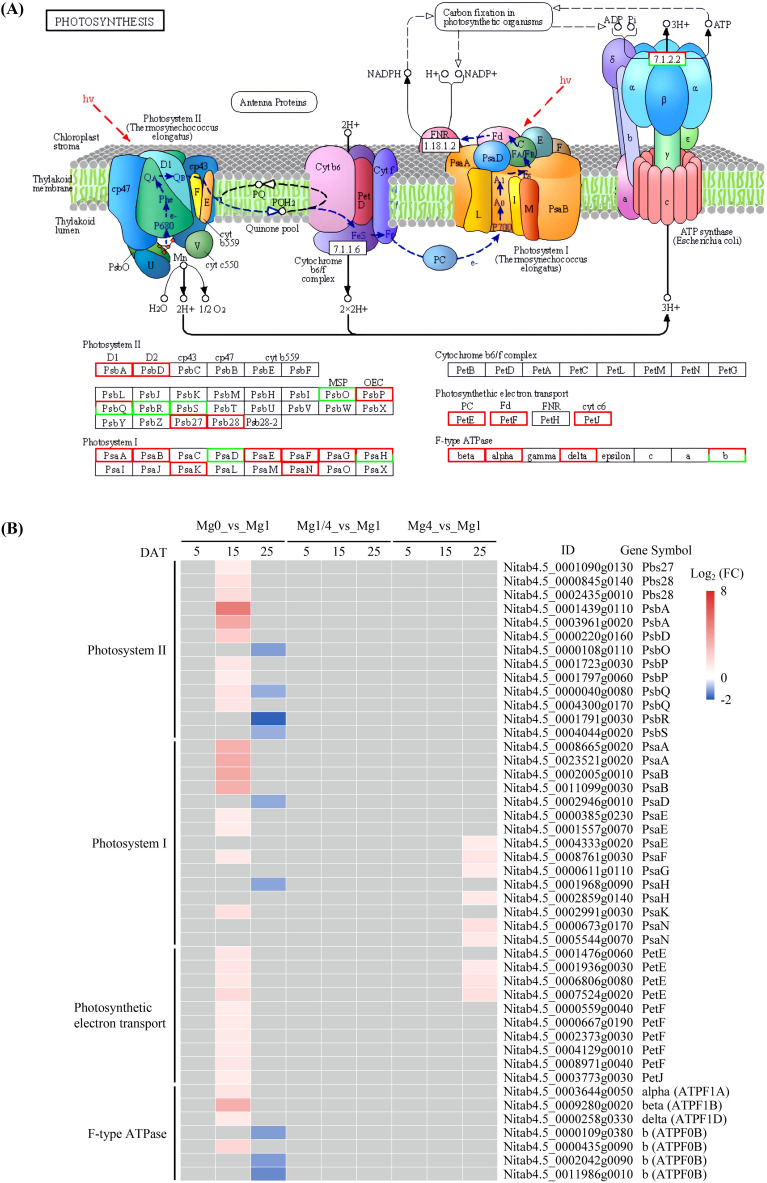
Expressional changes of KEGG-annotated DEGs in photosynthesis. **(A)** The KEGG pathway diagram showing DEGs involved in photosynthesis. The expression pattern for each gene is visualized as colors in the boxes containing the gene name. Red and green indicate that the genes were up-regulated and down-regulated, respectively. **(B)** Heatmap showing the differential expression patterns of DEGs involved in photosynthesis. Grey blocks indicate that the genes were not detected as DEGs by RNA-Seq. Mg0, 0 mM Mg; Mg1/4, 0.50 mM Mg; Mg1, 1.99 mM Mg; Mg4, 7.96 mM Mg. DAT, days after treatment.

The KEGG photosynthesis pathway was significantly enriched among the up-regulated DEGs in the Mg0 seedlings at 15 DAT, as well as among the down-regulated DEGs in the Mg0 seedlings and the up-regulated DEGs in the Mg4 seedlings at 25 DAT (**Supplementary Table S4**). The DEGs encoding proteins related to processes involved in the KEGG photosynthesis pathway, including photosystem II (PSII), photosystem I (PSI), photosynthetic electron transport, and F-type ATPase, were summarized (**Figure 4A**). A total of 45 DEGs related to the photosynthesis pathway were identified, with 32 up-regulated in the Mg0 seedlings at 15 DAT, nine down-regulated in the Mg0 seedlings at 25 DAT, and nine up-regulated in the Mg4 seedlings at 25 DAT (**Figure 4B**).

**Figure 5:**
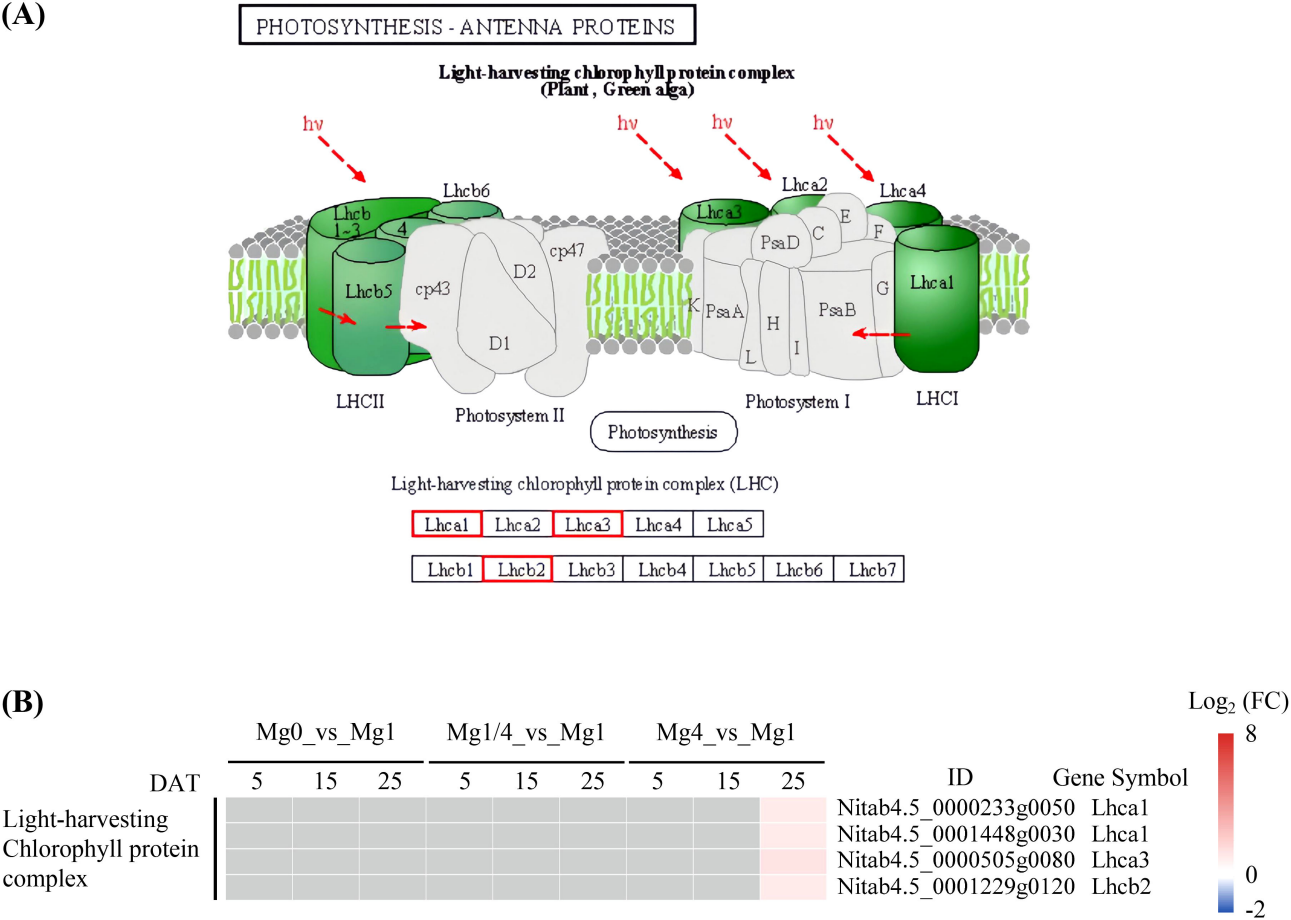
Expressional changes of KEGG-annotated DEGs in photosynthesis-antenna proteins. **(A)** The KEGG pathway diagram showing DEGs involved in photosynthesis-antenna proteins. The expression pattern for each gene is visualized as color in the boxes containing the gene name. Red indicates that the genes were up-regulated. **(B)** Heatmap showing the differential expression patterns of DEGs involved in photosynthesis-antenna proteins. Grey blocks indicate that the genes were not detected as DEGs by RNA-Seq. Mg0, 0 mM Mg; Mg1/4, 0.50 mM Mg; Mg1, 1.99 mM Mg; Mg4, 7.96 mM Mg. DAT, days after treatment.

**Figure 6:**
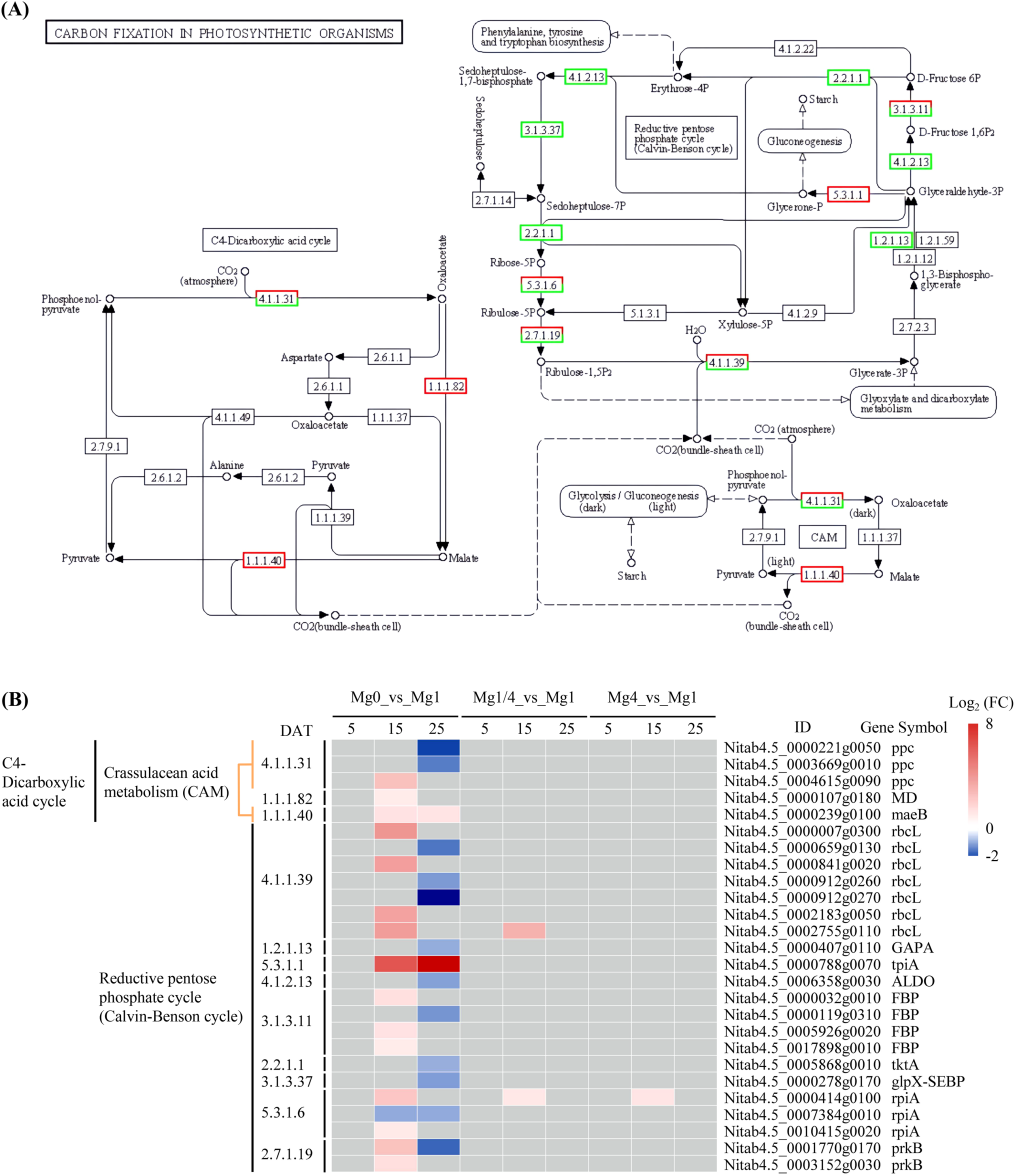
Expressional changes of KEGG-annotated DEGs in carbon fixation in photosynthetic organisms. **(A)** The KEGG pathway diagram showing DEGs involved in carbon fixation in photosynthetic organisms. The expression pattern for each gene is visualized as colors in the boxes containing the gene name. Red and green indicate that the genes were up-regulated and down-regulated, respectively. **(B)** Heatmap showing the differential expression patterns of DEGs involved in carbon fixation in photosynthetic organisms. Grey blocks indicate that the genes were not detected as DEGs by RNA-Seq. Mg0, 0 mM Mg; Mg1/4, 0.50 mM Mg; Mg1, 1.99 mM Mg; Mg4, 7.96 mM Mg. DAT, days after treatment.

The KEGG pathway of photosynthesis-antenna proteins was significantly enriched among the up-regulated DEGs in the Mg4 seedlings at 25 DAT (**Supplementary Table S4**). There were four DEGs related to the light-harvesting chlorophyll protein complex, all of which were up-regulated in the Mg4 seedlings at 25 DAT (**Figures 5A, B**). Additionally, the KEGG pathway of carbon fixation in photosynthetic organisms was significantly enriched among the up-regulated DEGs in the Mg0 seedlings at 15 DAT, and the down-regulated DEGs in the Mg0 seedlings at 25 DAT (**Supplementary Table S4**). A total of 26 DEGs related to processes (C4-Dicarboxylic acid cycle and reductive pentose phosphate cycle) involved in the carbon fixation in the photosynthetic organisms pathway were identified within this pathway. Of these, 15 DEGs were up-regulated in the Mg0 seedlings at 15 DAT, while 12 were down-regulated in the Mg0 seedlings at 25 DAT (**Figures 6A, B**).

### 3.5 DEGs involved in antioxidative regulation

**Figure 7:**
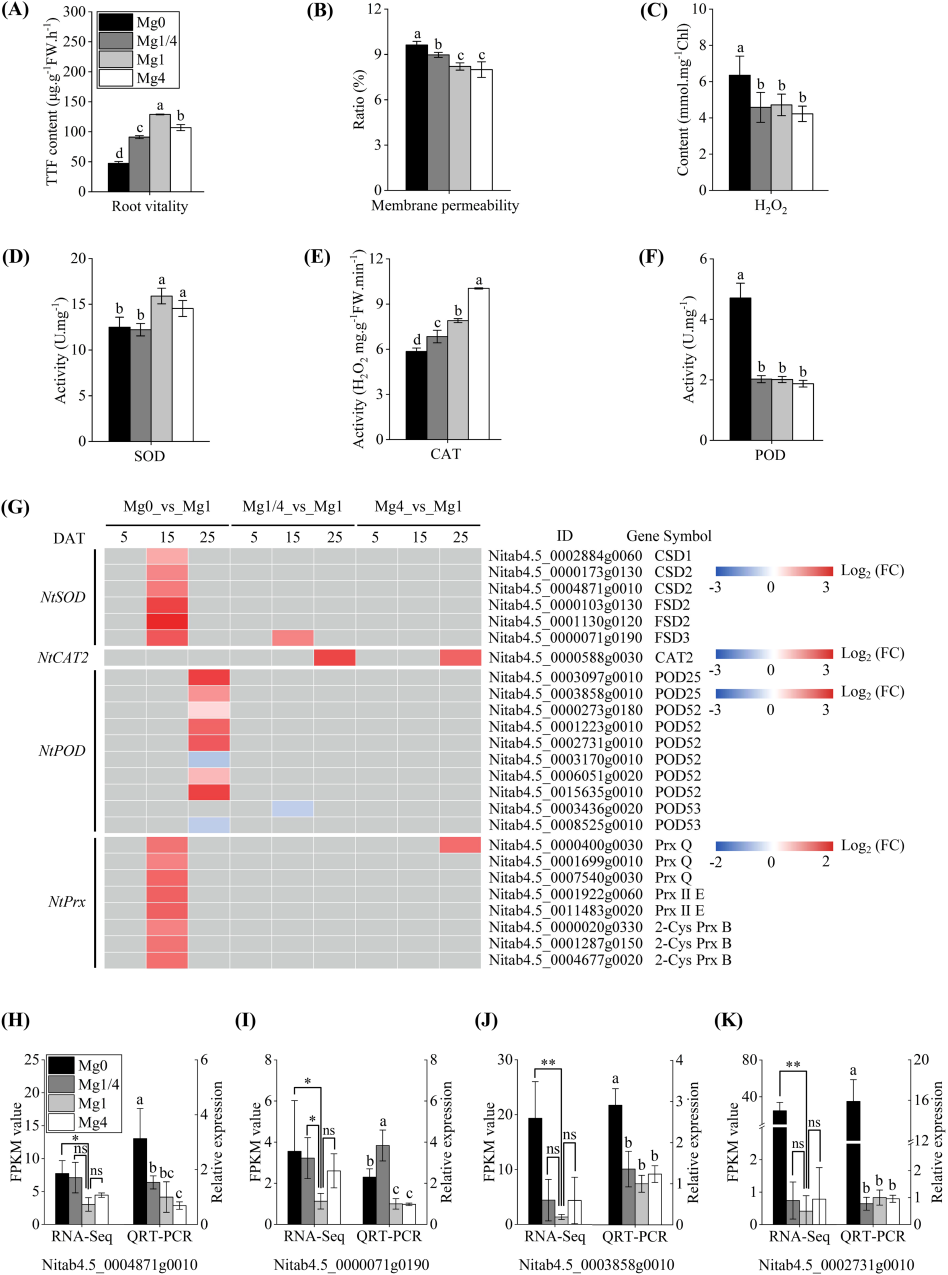
Effects of different Mg supplies on physiological parameters related to antioxidant defense and expressional changes of key DEGs involved in enzymatic scavenging. **(A-C)** Root vitality, Cell membrane permeability, and hydrogen peroxide (H_2_O_2_) content, respectively, in leaves of tobacco seedlings grown under different Mg supplies at 25 DAT. **(D-F)** Activities of superoxide dismutase (SOD), catalase (CAT), and peroxidase (POD) in leaves of tobacco seedlings grown under different Mg supplies at 25 DAT. **(G)** Heatmap showing the differential expression levels of SOD, CAT2, POD, and peroxiredoxin (Prx) family DEGs, respectively. Grey blocks indicate that the genes were not detected as DEGs by RNA-Seq. (**H-K**) qRT-PCR validation of two DEGs *NtCSD2* (*Nitab4.5_0004871g0010*) and *NtFSD3* (*Nitab4.5_0000071g0190*) in the Mg0, Mg1/4, Mg1, and Mg4 seedlings at 15 DAT, and two DEGs *NtPOD25* (*Nitab4.5_0003858g0010*) and *NtPOD52* (*Nitab4.5_0002731g0010*) in the Mg0, Mg1/4, Mg1, and Mg4 seedlings at 25 DAT. The tobacco *EF-1α* gene was used as an internal control. Mg0, 0 mM Mg; Mg1/4, 0.50 mM Mg; Mg1, 1.99 mM Mg; Mg4, 7.96 mM Mg. DAT, days after treatment. Significance levels *p < 0.05, **p < 0.01, ns, not significant. Different letters (a, b, c, d) above the columns indicate statistical differences (p < 0.05).

Plant leaves that are deficient in Mg are highly photosensitive, leading to an over-saturation of photosynthetic electron transport system. Under highly reduced condition, electrons could pass-on to O_2_, resulting in the generation of O_2_^-^ and other reactive oxygen species (ROS) (Grossman and Takahashi, 2001). At 25 DAT, the root vitality (measured by the triphenylmethyl hydrazone (TTF) content), the relative level of CMP, H_2_O_2_ content, and the activities of the enzymatic scavengers SOD, POD, and CAT in leaves of tobacco seedlings grown under different Mg levels were measured. The root vitality was significantly lower in the Mg0 seedlings compared to that in the Mg1/4, Mg1, and Mg4 seedlings (**Figure 7A**), and the CMP level and H_2_O_2_ content were notably higher in the Mg0 seedlings compared to the others (**Figures 7B, C**). These results indicate that Mg deficiency cause oxidative stress in tobacco seedlings. As for antioxidant enzymes, the activity of SOD was significantly lower in the Mg0 seedlings compared to the Mg1 and Mg4 seedlings (**Figure 7D**). Similarly, CAT activity was notably lower in the Mg0 seedlings compared to the Mg1/4, Mg1, and Mg4 seedlings (**Figure 7E**). In contrast, POD activity in the Mg0 seedlings were significantly higher than that in the Mg1/4, Mg1, and Mg4 seedlings (**Figure 7F**).

**Figure 8:**
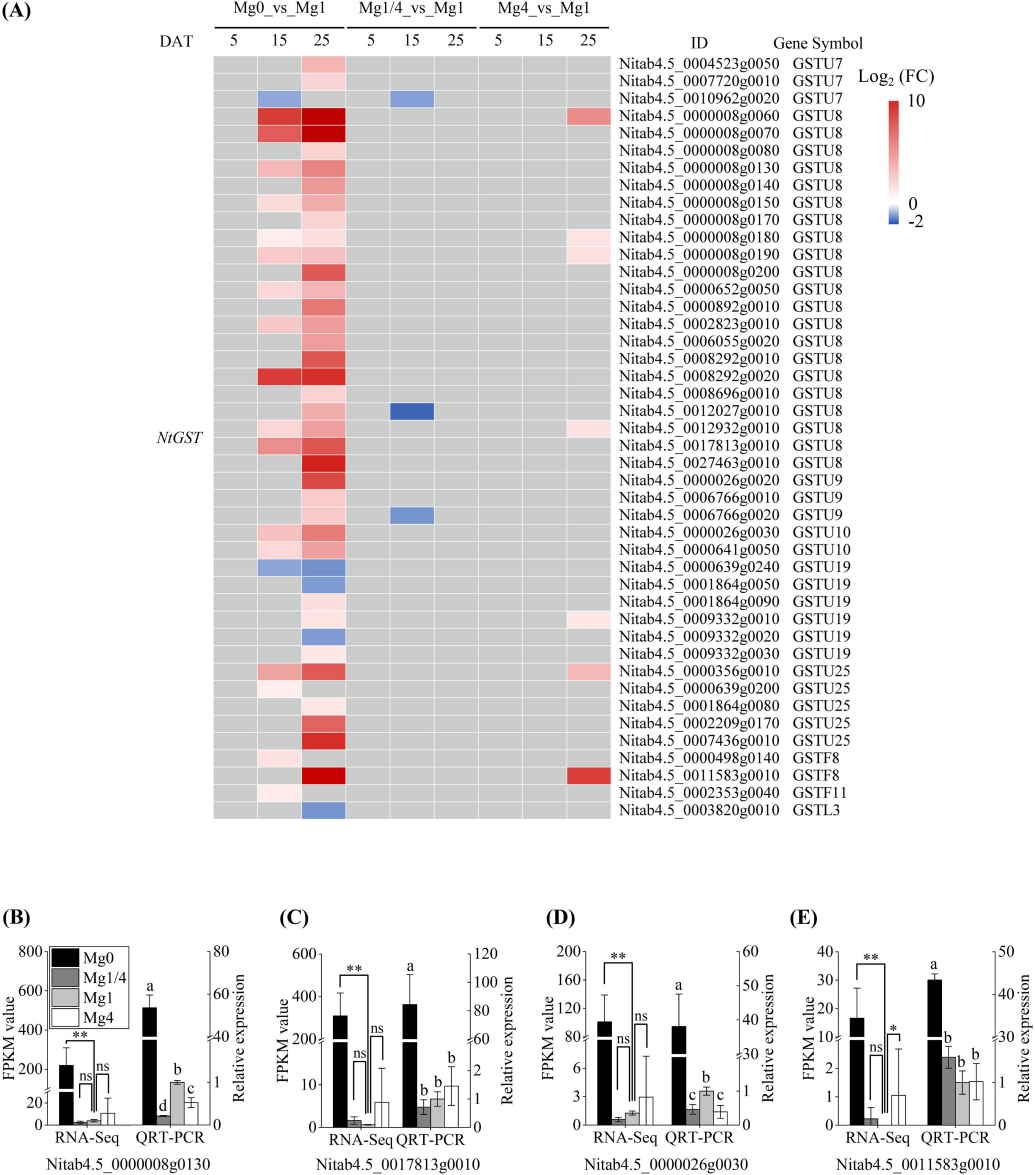
Expressional changes of glutathione S-transferase (GST) family DEGs. **(A)** Heatmap showing the differential expression levels of GST family DEGs in leaves of tobacco seedlings grown under different Mg supplies. Grey blocks indicate that the genes were not detected as DEGs by RNA-Seq. **(B-E)** qRT-PCR validation of four DEGs *NtGST8* (*Nitab4.5_0000008g0130*), *NtGST8* (*Nitab4.5_0017813g0010*), *NtGST10* (*Nitab4.5_0000026g0030*), and *NtGSTF8* (*Nitab4.5_0011583g0010*) in the Mg0, Mg1/4, Mg1, and Mg4 seedlings at 25 DAT. The tobacco *EF-1α* gene was used as an internal control. DAT, days after treatment. Significance levels *p < 0.05, **p < 0.01, ns, not significant. Different letters (a, b, c, d) above the columns indicate statistical differences (p < 0.05).

We further characterized the DEGs related to antioxidant responses. Six *SOD* homologous genes were identified among the DEGs, including three copper/zinc superoxide dismutase genes (*CSDs*) and three iron superoxide dismutase genes (*FSDs*) (**Figure 7G**). Surprisingly, despite the lower SOD activity detected in the Mg0 seedlings at 25 DAT, all six *SOD* homologous genes were found to be up-regulated in the Mg0 seedlings at 15 DAT. Furthermore, one *CAT2* homologous gene (*Nitab4.5_0000588g0030*) was identified as a DEGs that was up-regulated in the Mg1/4 and Mg4 seedlings at 25 DAT (**Figure 7G**). Additionally, 10 *POD* homologous genes were identified among the DEGs, with the majority (seven out of 10) being up-regulated in the Mg0 seedlings at 25 DAT (**Figure 7G**), consistent with the significantly higher POD activity detected in the Mg0 seedlings at 25 DAT. Moreover, eight *peroxiredoxin* (*Prx*) homologous genes were identified among the DEGs, including three *peroxiredoxin Q* genes (*Prx Q*), two *peroxiredoxin II E* genes (*Prx II E*), and three *2-Cys peroxiredoxin B* genes (*2-Cys Prx B*). All eight *Prx* homologous genes were up-regulated in the Mg0 seedlings at 15 DAT (**Figure 7G**). The transcriptional profiles of the DEGs *NtCSD2* (*Nitab4.5_0004871g0010*) and *NtFSD3* (*Nitab4.5_0000071g0190*) in the Mg0, Mg1/4, Mg1, and Mg4 seedlings at 15 DAT, and *NtPOD25* (*Nitab4.5_0003858g0010*) and *NtPOD52* (*Nitab4.5_0002731g0010*) in the Mg0, Mg1/4, Mg1, and Mg4 seedlings at 25 DAT were validated using real-time RT-PCR. The results showed a significant up-regulation of these four genes in the Mg0 seedlings compared to the Mg1 seedlings, and the results were in line with the RNA-Seq data (**Figures 7H-K**). In addition to the *SOD*, *CAT2*, *POD*, and *Prx* homologous genes, a total of 44 glutathione S-transferase (GST) homologous genes were identified among the DEGs. These included three *NtGSTU7*, 21 *NtGSTU8*, three *NtGSTU9*, two *NtGSTU10*, six *NtGSTU19*, five *NtGSTU25*, two *NtGSTF8*, one *NtGSTF11*, and one *NtGSTL3* (**Figure 8A**). The majority of these *GST* homologous genes (39 out of 44) were up-regulated in the Mg0 seedlings at 15 and/or 25 DAT. The transcriptional profiles of the DEGs *NtGST8* (*Nitab4.5_0000008g0130*), *NtGST8* (*Nitab4.5_0017813g0010*), *NtGST10* (*Nitab4.5_0000026g0030*), and *NtGSTF8* (*Nitab4.5_0011583g0010*) in the Mg0, Mg1/4, Mg1, and Mg4 seedlings at 25 DAT were validated by real-time RT-PCR. The results showed a significant up-regulation of these four *NtGST* homologous genes in the Mg0 seedlings, consistent with the RNA-Seq data (**Figures 8B-E**).

## 4 Discussion

Mg is a vital element essential for plant growth and development. Both insufficient and excessive supply of Mg can adversely affect the growth, development, and productivity of various crops. In this study, we performed a comprehensive transcriptome analysis to explore the molecular responses of tobacco seedlings to varying levels of Mg supply. The analysis revealed significant changes in gene expression in tobacco leaves, particularly under conditions of Mg deficiency.

**Figure 9:**
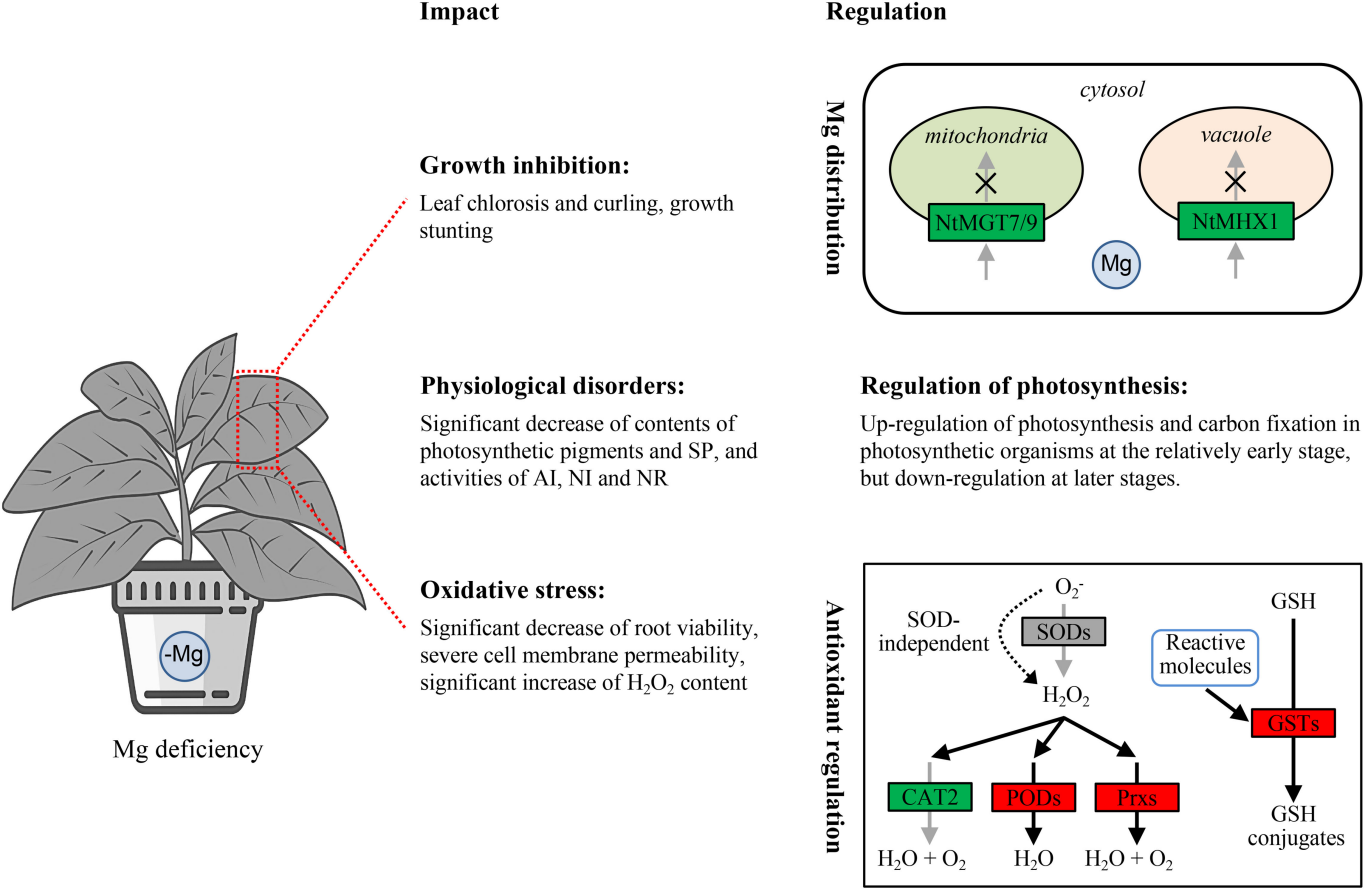
Schematic model of the molecular regulation mechanisms of Mg distribution, photosynthesis regulation and antioxidant regulation underlying the response to Mg deficiency in leaves of tobacco seedlings. SODs, superoxide dismutases; CAT2, catalase; PODs, peroxidases; Prxs, peroxiredoxins; GSH, glutathione; GSTs, glutathione S-transferases. Red and green backgrounds indicate that the genes were up-regulated and down-regulated, respectively.

Plants have evolved strategies to regulate cellular Mg homeostasis. Studies on *Arabidopsis* and many other plants have revealed that MGTs and MHXs are crucial for uptake and distribution of Mg. In *Arabidopsis*, AtMGT1 and AtMGT10 have been demonstrated to be capable of transporting Mg^2+^ (Li et al., 2001). AtMGT1 and AtMGT2 are localized in the tonoplast and facilitate mobilizing Mg^2+^ into vacuole (Tang et al., 2022). AtMGT3 has been identified as being involved in regulating Mg homeostasis in mesophyll cells (Alexandersson et al., 2004; Whiteman et al., 2008). AtMGT5 is localized in the mitochondria and functions as a Mg-importer at low micromolar levels while facilitating Mg efflux at higher millimolar concentrations (Li et al., 2008). AtMGT6 and AtMGT7 are also involved in regulating cellular Mg^2+^ homeostasis. The mutation or knockdown of *AtMGT6* and/or *AtMGT7* result in Mg^2+^ hypersensitivity (Gebert et al., 2009; Mao et al., 2014; Oda et al., 2016). MGTs are present in the plasma membrane, mitochondria, tonoplast, endoplasmic reticulum, and chloroplast (Oda et al., 2016), indicating their role in mediating Mg movement between the cytosol and organelles. In this study, two homologous *MGTs* were identified as DEGs, including *NtMGT7* and *NtMGT9*. These two genes were down-regulated in tobacco seedlings under Mg deficiency at 25 DAT (**Figure 3A**). While many *MGTs* were identified that are up-regulated in plants under Mg starvation in previous studies (Hermans et al., 2010; Mao et al., 2014; Li et al., 2016; Ge et al., 2022; Bin et al., 2023), our study did not find significantly up-regulated homologous *MGTs* in the Mg0 seedlings. This could be due to using only leaves for RNA-Seq analysis in the present study. Our research further demonstrated that the homologous NtMGT7 and NtMGT9 are localized in the mitochondria (**Figure 3F**), suggesting their involvement in Mg transport between the cytosol and mitochondria. MHX functions as a proton exchanger responsible for Mg^2+^ transport across the vacuolar membrane (Berezin et al., 2008; Kobayashi, 2022). In the present study, a homologous *NtMHX1* gene was identified as a DEG. Similar to *NtMGT7 and NtMGT9*, *NtMHX1* was down-regulated in tobacco seedlings under Mg deficiency at 25 DAT (**Figure 3B**). Consistent with the localization of AtMHX1 in the vacuole membrane of *Arabidopsis* cells (Conn et al., 2011), the homologous NtMHX1 is localized in the vacuole membrane of *N. benthamiana* cells (**Figure 3F**). Overall, these results suggest a down-regulation of Mg-trafficking from the cytosol to mitochondria and vacuole in the leaf cells of tobacco seedlings experiencing Mg deficiency (**Figure 9**).

It is widely acknowledged that Mg deficiency inhibits photosynthesis in various plant species, such as Citrus (Yang et al., 2012), watermelon (Huang et al., 2016), barley (Jaghdani et al., 2021a), *Spinacia oleracea* (Jaghdani et al., 2021b), cucumber (Meng et al., 2023), and rice (Zhou et al., 2024). Mg is essential for chlorophyll formation. In the present study, significantly lower levels of Chla and Chlb were observed in the Mg0 seedlings compared to those supplied with varying levels of Mg (Mg1/4, Mg1, and Mg4), indicating a decline of chlorophyll formation caused by Mg deficiency. KEGG analysis revealed that the pathways of photosynthesis and carbon fixation in photosynthetic organisms were significantly enriched among the up-regulated DEGs in the Mg0 seedlings at 15 DAT, but among the down-regulated DEGs in the Mg0 seedlings at 25 DAT (**Figures 4B**, **6B**). A total of 45 DEGs related to PSII, PSI, photosynthetic electron transport, and F-type ATPase were identified, with 32 being up-regulated in the Mg0 seedlings at 15 DAT, and nine down-regulated in the Mg0 seedlings at 25 DAT (**Figure 4B**); furthermore, a total of 26 DEGs related to C4-Dicarboxylic acid cycle and reductive pentose phosphate cycle were identified, with 15 up-regulated in the Mg0 seedlings at 15 DAT, and 12 down-regulated in the Mg0 seedlings at 25 DAT (**Figures 6A, B**). These results suggest that Mg deficiency initially triggers the up-regulated expression of genes related to photosynthesis and carbon fixation in tobacco seedlings at relatively earlier stages, followed by down-regulation at later stages (**Figure 9**). The down-regulation of genes related to photosynthesis and carbon fixation at relatively late stages aligns with previous findings showing that Mg deficiency inhibits plant photosynthesis (Yang et al., 2012; Huang et al., 2016; Jaghdani et al., 2021a; b; Meng et al., 2023; Zhou et al., 2024).

Deficiencies in macronutrient or micronutrient elements can lead to oxidative stress in plants. For example, Zn deficiency can cause severe cell membrane damage and increased H_2_O_2_ levels in tobacco seedlings (Lu et al., 2023). Similarly, Mg deficiency can impair the electron transport rate, leading to an over-reduction of the electron transport chain, ultimately triggering the production of ROS (Hermans and Verbruggen, 2005; Tang et al., 2012). In the present study, it was observed that root vitality was significantly reduced, and the levels of cell membrane damage (as indicated by CMP) and H_2_O_2_ were significantly higher in leaves of tobacco seedlings experiencing Mg deficiency (**Figures 7A-C**), indicating the induction of oxidative stress (**Figures 7A-C**). Six *NtSOD* family DEGs were up-regulated in the Mg0 seedlings at 15 DAT (**Figure 7G**). Conversely, reduced SOD activity was observed in tobacco seedlings under Mg starvation at 25 DAT (**Figure 7D**). This reduction in SOD activity could be due to metabolic disorders caused by Mg deficiency or other unknown reasons. Additionally, the finding suggests the potential dismutation of oxide ion (O_2_^-^) through an SOD-independent mechanism in tobacco seedlings under Mg deficiency (**Figure 9**). Similarly, decreased CAT activity was observed in tobacco seedlings experiencing Mg starvation at 25 DAT (**Figure 7E**). In contrast, significantly enhanced POD activity was detected in the Mg0 seedlings at 25 DAT (**Figure 7F**). Consistent with this increased activity, seven *NtPOD* homologous genes were up-regulated in leaves of tobacco seedlings under Mg deficiency (**Figure 7G**). Moreover, eight DEGs encoding Prxs, a specific class of H_2_O_2_-decomposing antioxidant enzymes (Smirnoff and Arnaud, 2019), were up-regulated in the Mg0 seedlings at 15 DAT (**Figure 7G**). The up-regulation of these *NtPODs* and *NtPrxs* DEGs is expected to facilitate ROS scavenging (**Figure 9**). Furthermore, a total of 44 *NtGST* homologous genes were identified as DEGs, with 39 out of the 44 *NtGST* DEGs being up-regulated in response to Mg deficiency at 15 and/or 25 DAT (**Figure 8A**). Similarly, the up-regulation of *NtGSTs* would contribute to maintaining ROS homeostasis (**Figure 9**).

## 5 Conclusions

Our results demonstrated that Mg deficiency caused severe physiological disorders and inhibits the growth of tobacco seedlings. The global gene expression profiles revealed potential mechanisms involved in the response to Mg deficiency in tobacco leaves. These mechanisms include the down-regulation of genes associated with Mg trafficking from the cytosol to mitochondria and vacuoles, the down-regulation of genes related to photosynthesis and carbon fixation at later stages, and the up-regulation of genes related to antioxidant defenses.

## Data Availability

The datasets presented in this study can be found in online repositories. The names of the repository/repositories and accession number(s) can be found in the article/**Supplementary Material**.

## Supplementary material

Supplementary Table 1 Primers used in this study.

Supplementary Table 2 Total DEGs in Mg0, Mg1/4, and Mg4 in comparison to Mg1.

Supplementary Table 3 DEGs in Mg0, Mg1/4, and Mg4 in comparison to Mg1 at 5, 15, and 25 DAT, respectively.

Supplementary Table 4 Significant KEGG pathways of DEGs in Mg0, Mg1/4, and Mg4 in comparison to Mg1.
